# The bacterial promoter spacer modulates promoter strength and timing by length, TG-motifs and DNA supercoiling sensitivity

**DOI:** 10.1038/s41598-021-03817-4

**Published:** 2021-12-22

**Authors:** Carlo A. Klein, Marc Teufel, Carl J. Weile, Patrick Sobetzko

**Affiliations:** SYNMIKRO, Center for Synthetic Microbiology, Philipps-Universität Marburg, Hans-Meerwein-Straße 6, 35043 Marburg, Hesse Germany

**Keywords:** Gene regulation, Bacterial synthetic biology, Bacterial genetics

## Abstract

Transcription, the first step to gene expression, is a central coordination process in all living matter. Besides a plethora of regulatory mechanisms, the promoter architecture sets the foundation of expression strength, timing and the potential for further regulatory modulation. In this study, we investigate the effects of promoter spacer length and sequence composition on strength and supercoiling sensitivity in bacteria. Combining transcriptomics data analysis and standardized synthetic promoter libraries, we exclude effects of specific promoter sequence contexts. Analysis of promoter activity shows a strong variance with spacer length and spacer sequence composition. A detailed study of the spacer sequence composition under selective conditions reveals an extension to the -10 region that enhances RNAP binding but damps promoter activity. Using physiological changes in DNA supercoiling levels, we link promoter supercoiling sensitivity to overall spacer GC-content. Time-resolved promoter activity screens, only possible with a novel mild treatment approach, reveal strong promoter timing potentials solely based on DNA supercoiling sensitivity in the absence of regulatory sites or alternative sigma factors.

## Introduction

For long-term survival in its niche, bacteria need to adapt their transcription pattern to the overall conditions met. Prokaryotes can modulate transcription in multiple ways, either by changing the affinity of the RNA-polymerase holoenzyme (RNAP) to the promoter or by preventing or enhancing the binding to the promoter by additional regulators. However, all transcriptional regulation relies on the basal features of the promoter that are in turn modulated in strength and timing by additional regulatory mechanisms coupled to internal and external requirements. Although there are variations for specialized sigma factors, the promoter structure has defined regions upstream of the first transcribed base (+1): the discriminator, $-10$-region, the spacer, $-35$-region and the UP-element. The $-10$ region, together with the $-35$-sequence and parts of the spacer, interacts with the sigma factor of the RNA-polymerase holoenzyme. The housekeeping sigma factor $\sigma {}$^70^ binds about 80% of the currently known promoters in *E. coli*.The $-10$ and the $-35$ regions are separated by the spacer. In *E. coli* promoters, the spacer has a flexible length. The most abundant (optimal) spacer is 17 bp ± 1 bp in length^1^. The sequence of the spacer region is rarely conserved. Nevertheless, previous promoter studies have shown that the spacer sequence composition can impact promoter activity^2^. However, in the 1980s, a conserved TG-motif at position -16 was found in *Bacillus subtilis* and later in gram-negative as well as in gram-positive bacteria^3–5^. In *E. coli*, this motif occurs in roughly 25% of the promoters and, due to its proximity to the $-10$ region, was named the ’extended $-10$’^6^. Mainly promoters with no or a weak $-35$ sequence had this TG-motif, and thus it was inferred that the TG-motif compensates for the lack of the $-35$ region by interacting with the region 3.0 of the sigma factor^7,8^. Further upstream of the $-35$ region, an AT-rich area of around 40 bp was shown^9^. This so-called ’UP-element’ can interact with the alpha subunit of the RNAP and thus can increase the affinity of RNAP to the promoter^10^. It is predominantly found in strong ribosomal RNA and protein promoters. The discriminator, a second 6 bp ± 1 bp long spacing element between the first transcribed base (+1) and the $-10$-region, is responsible for influencing the melting of the DNA-double-helix during open-complex formation. Recent studies provided strong evidence for its role in DNA supercoiling sensitivity^11^. Together, the described promoter elements bind the RNAP-holoenzyme and thereby modulate the ensuing expression strength of the downstream coding sequence according to the mix and match model^12,13^. For native promoters, the basal promoter structure is interwoven with additional regulatory sites to bind proteins that modulate expression strength and timing. However, already for the basal promoters, regulation is possible in the absence of regulatory sites. Here, DNA supercoiling is a major regulator exploiting fundamental properties of the DNA double helix. The topological stress trapped in the DNA helix can facilitate or impede DNA unwinding during open complex formation^14^. In *Escherichia coli*, DNA supercoiling levels are tightly regulated by four topoisomerases. The most prominent ones, Gyrase and Topoisomerase I, are coupled in an antagonistic way to control DNA supercoiling levels throughout the growth cycle^15,16^. Further regulation is achieved by buffering of free DNA supercoils via abundant nucleoid-associated proteins (NAPs) constraining DNA supercoils and removing them from the pool of Topoisomerase susceptible supercoils^17,18^. Hence, the activity of topoisomerases in combination with the abundance of various NAPs determines the overall free DNA supercoiling level in the cell. By nutrient and stress-dependent changes in NAP abundance and topoisomerase activity, DNA supercoiling levels change throughout the bacterial growth cycle^19^. Except for some extremophile species, overall DNA supercoiling is negative throughout the bacterial growth cycle^20,21^. During Lag-phase towards early exponential phase DNA supercoiling reaches its most negative state, which facilitates open-complex formation of stable RNA promoters and other highly transcribed genes, essential during fast growth^16,22^. From late exponential phase through transition phase and into stationary phase, DNA supercoiling levels relax, which in turn supports the shutdown of exponential phase promoters. With almost half of the genes sensitive to changes in DNA supercoiling, it is a major regulator of growth dependent cellular processes^16^. In addition to control of overall DNA supercoiling levels by Topoisomerases and NAPs, transcription has an impact on local DNA supercoiling levels. According to the Liu and Wang twin-supercoiled-domain model, transcription activity alters DNA supercoiling around the transcription complex in an asymmetric manner^23^. During transcription, the template DNA faces torsional stress. Previous studies have shown that this transcription-coupled supercoiling (TCDS) transmits 10–15 kb up- and downstream of the transcription site and is present across the genome^24–26^. TCDS is observed in prokaryotes as well as in eukaryotes^27^ and has similar overall effects on the chromatin organisation^28^. Furthermore, TCDS affects transcription levels of nearby genes, depending on their supercoiling preference and position relative to the emitting gene^29^. Therefore, TCDS is a chromosome shaping factor, influencing preferred gene arrangement on an evolutionary time scale^27,30–33^. However, the question of how the promoter itself can be responsive to supercoiling remains. One promoter element that influences the topology of the constituent parts of the promoter which interacts with the RNAP is the spacer. A spacer length of 17 bp is placing the centres of the two motifs $-35$ and $-10$ almost two helical turns of B-DNA apart^34^. Consequently, it was shown for single promoters that the variation of the spacer length modulates the response of a promoter to DNA supercoiling^35^, thus making the spacer a *bona fide* target for a comprehensive study. In this study, we present an analysis of hundreds of native promoters as well as synthetic core promoters with various spacer lengths and sequences. We show that spacer length and sequence composition affect DNA supercoiling sensitivity and promoter strength significantly. Furthermore, using selective conditions, we identify novel sequence motifs within the spacer sequence.

## Materials and methods

### Transcriptomics analysis

The 50 bp reads from Sobetzko et al. 2013^31^ were mapped on the *E. coli* MG1655 genome (NCBI) using the R package QuasR. Gene expression was determined by normalizing the CDS reads by the total number of reads as well as the length of the CDS. The resulting expression values sums up to 1 for all genes. To make the numbers more intuitive than Reads Per Kilobase of transcript, per Million mapped reads (RPKM), we divided the expression values by the average expression of all genes. Hence, the resulting relative expression values indicate the fold difference of the gene expression to the average gene expression. E.g, a value of 10 indicates a 10 times higher expression that the average gene. Maximal expression of each gene in the time-resolved expression data was used to define the promoter strength. Promoter features of RegulonDB version 10.5 (http://regulondb.ccg.unam.mx/) were used to extract promoter spacer length and sequence. $\sigma {}$^70^ promoters were filtered by a maximum of 3 bases deviation from the consensus sequence to reduce influence of the $-10$ and $-35$ region features on expression data and achieve maximum compatibility with the synthetic library data.

### Promoter library preparation

For the Golden Gate^36^ insertion of the spacer sequences, 12 oligos for different spacer lengths and one complementary oligo to generate dsDNA of the 12 oligos were used. The first 12 oligos contained the full length random sequence (Ns) of the spacer sequence with restriction sites for BsaI and a sequence for binding of a 13th complementary oligo to generate double-stranded DNA fragments by annealing and DNA Polymerase extension (5’-**GGTCTC**GGACA(N)$_{n}$TATA$\underline{\hbox {C}\mathbf{GAGACC}\hbox {GTGTCTATCAC}}$-3’, 5’-GTGATAGACACGGTCTCG-3, n = 12 to n = 23, BsaI sites in bold letters, complementary region underlined). The resulting double-stranded spacer DNA fragments were purified by a commercial DNA purification kit (Roth). At this stage, each DNA molecule in the library contained a different spacer sequence. For the insertion, equimolar amounts (40nM) of the spacer fragments and the vector backbone were mixed and supplemented with Ligase buffer, 200 units Ligase and 10 units BsaI-HF®v2 (NEB). The mix was incubated for 5 h at 37 °C and transformed using Top10 cells. Unmodified vectors containing the Gyrase inhibiting ccdB gene and lacZ alpha fragment in the insertion site were selected against by ccdB toxicity and detected by alpha complementation. Hence, all cells containing unmodified vector would either die or show a blue color if ccdB selection fails.

### Promoter library analysis

In all experiments, cells were grown in LB at 37 °C on a plate shaker at 200 rpm. OD600 and Fluorescence measurements were performed in a Infinite 200 PRO plate reader (Tecan). OD600 as well as fluorescence signals (RFU) were normalized to a blank (medium only). RFU/OD600, indicating the relative cellular concentration of the fluorescence signal, was determined from normalized OD600 and fluorescence signals.

### DNA supercoiling sensitivity studies

Novobiocin is known to inhibit DNA gyrase and consequently relaxes the DNA in vivo^37–39^. To investigate the relationship between DNA-relaxation and promoter strength, we used sub-lethal Novobiocin concentrations (17 µg/µL). At the applied concentration no growth defect was detected throughout the growth cycle (Fig. 6C,D). At higher concentrations above 20 17 µg/μL, not applied in this study, the expected growth defects of the gyrase inhibitor are observed (data not shown). For the analysis 96 fresh colonies were picked for each spacer length from library plates and inoculated overnight in LB in a 96-well plate. Colonies were picked from several transformations and on each of the 12 96-well plates, 8 of each spacer length (12 spacer x 8 colonies) were present to prevent transformation and plate related biases. The next day, the plate was copied to two fresh plates with a dilution factor of 1:100, one plate contained 100 µL LB + antibiotic, and the other one additional 17 µg/µL novobiocin. Finally, we added 50 µL mineral oil to avoid evaporation effects. Both plates were incubated 15 h at 37 °C on a plate shaker at 200 rpm. The next day, OD600 and fluorescence were measured in an Infinite 200 PRO plate reader (Tecan). OD600 and fluorescence was normalized to blanks. Fluorescence was further normalized by the normalized OD600 and compared between treated and untreated samples. Clones of interest were confirmed by Sanger sequencing.

### EMSA assay

To measure the interaction between a protein and a DNA fragment, Electrophoretic Mobility Shift Assay (EMSA) was performed. The DNA template comprised a 150 bp fragment amplified from the respective plasmids using cy3-labelled primer ( 5’-CTCTGGCGAAGACATGGAG-3’ and GACCAGGATGGGCACCACCC) . The promoter region was centered on the fragment. DNA was extracted and tested for purity by native PAGE. First, a 5 % polyacrylamide gel was prepared. The RNAP$\sigma ^{70}$ holoenzyme (NEB) and cy3-labelled double stranded DNA was diluted in RNAP reaction buffer (NEB) to the final concentrations used in the assay (40 mM Tris-HCl, 150 mM KCl, 10 mM MgCl, 21 mM dithiothreitol, 0.01% Triton X-100). The binding reaction was setup to a reaction volume of 10 µL at 20 °C in the dark for 30 min with a DNA fragment concentration of 4 nM. Polyacrylamide Gel Electrophoresis (PAGE) was prerun for 10 min in 0.5 % TBE buffer at 10 V. Immediately before loading, 2.5 µL SDS-free loading buffer was added to the binding reaction and 10 µL were applied to each well. The PAGE was run at 10 V for 10 min and then shifted to 60 V for another hour. Gel images were captured with a Typhoon fluorescence scanner. Background subtraction and quantification was done with the open source imageJ software. Data visualization and regression was done with a custom R script using non-linear regression of the Hill equation where $\theta$ is the fraction of the DNA-polymerase concentration that is bound by the DNA, $K_D$ is the dissociation constant, n is the Hill-coefficient and P is the total DNA-polymerase concentration.

1 $$\begin{aligned} \theta =\frac{[P]^n}{K_D^n+[P]^n} \end{aligned}$$

## Results

### Promoter spacer length and sequence has a strong impact on promoter strength

**Figure 1:**
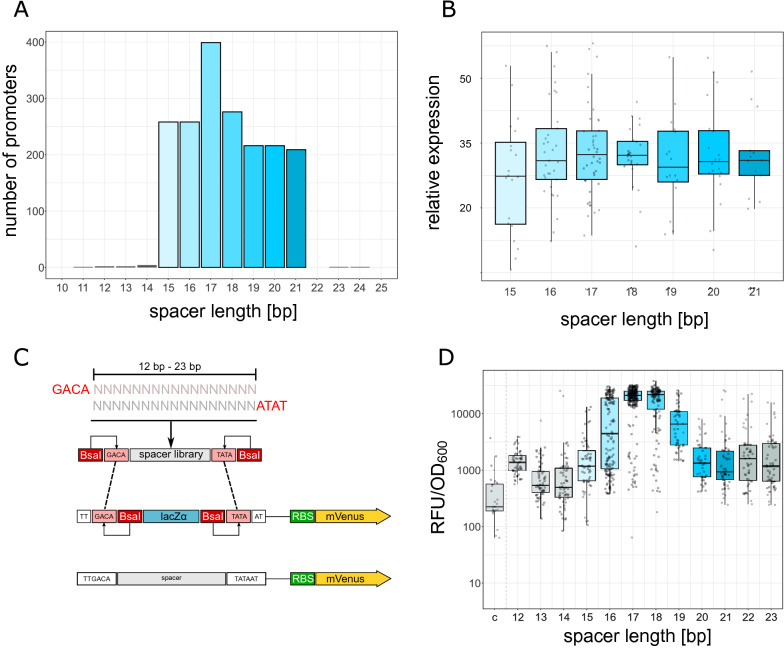
Impact of spacer length and spacer sequence composition on promoter strength. **(A)** Frequency of native *Escherichia coli* $\sigma {}$^70^ promoter spacer lengths. **(B)** RNA-seq expression data of *Escherichia coli* $\sigma {}$^70^ promoter driven genes with no more than 3 mismatches in total to the consensus of $-10$ and $-35$ region. As in RNA-seq no absolute amounts of cellular transcripts can be determined, relative expression is used. The ordinate scale indicates the fold change of expression relative to the average gene expression. **(C)** Cloning scheme of the synthetic promoter library. Golden Gate cloning is used to insert the spacer library into the p15A-cmR plasmid backbone. **(D)** Fluorescence signal (mVenus) of the synthetic promoter libraries normalized by OD$_{600}$. Blue colors indicate the native spacer range. Grey colors indicate spacer lengths outside of the native range. The ’c’ column represents fluorescence signals with the promoter region replaced by random DNA sequence library to estimate background or read-through signals.

Native promoter strength can vary over several orders of magnitude between stable RNA promoters and promoters for genes with lowest expression in bacteria. This wide range provides a first level of control of the cellular stoichiometry of RNAs and proteins. Early studies suggested that spacer length can have an impact on promoter strength. These studies relied on single promoter studies with a limited set of specific modifications. To avoid conclusions based on specific promoter sequence contexts, we investigated the diverse set of curated *Escherichia coli* $\sigma {}$^70^-promoters annotated in the regulonDB database http://regulondb.ccg.unam.mx/^40^. Data analysis revealed a spacer length distribution between 15 bp and 21 bp for endogenous $\sigma {}$^70^-promoters (Fig. 1A, see also Fig. S7 for all promoters). Further analysis of the corresponding expression using high-throughput transcriptomics data^31^ showed a statistically non-significant but declining trend of average promoter strength from an optimal spacer length of 17/18 bp (Fig. 1B). The 17 bp spacer with the highest average expression values is also the most prominent spacer architecture followed by the 18 bp spacer with the second highest average expression values (Fig. 1A,B). Promoter strength not only differs between spacer lengths, but also within a group of promoters with identical spacer length, indicating a contribution of spacer sequence composition to promoter strength. However, transcriptomics data comprise highly diverse endogenous promoter including multiple impact factors such as regulatory sites, DNA supercoiling sensitivity, variations in the -35 and -10 regions as well as variations in the discriminator and up-elements. This complexity may hide or bias the spacer length effects. To isolate the impact of the promoter spacer element, we followed a plasmid-born synthetic approach with uniform sequence context and promoter architecture. The natural limits of spacer length from 15 bp to 21 bp were extended from 12 bp to 23 bp to investigate effects of non-native spacer lengths. Using a random oligo library (see methods) cloned into a plasmid-born consensus $\sigma {}$^70^ promoter backbone, promoters of various spacer length and random spacer sequence were generated (Fig. S8). The spacer sequence space was set to the full spectrum using only Ns during oligo synthesis. This allows for unbiased library composition. The formation of random promoters was tested by replacing the promoter region by a random sequence only containing Ns (Fig. 1D ’c’ column). We also checked the library integrity by cloning into a non-promoter context and verified homogeneous nucleotide distribution by Sanger sequencing. For the promoter sequence context of the vector, no apparent RNAP binding signals were present and the context remained constant throughout the whole study. Furthermore, a highly efficient upstream artificial terminator prevented potential read-through by other transcribed elements on the vector (see supplementary vector map). Promoter activity was determined by a fluorescence signal of the coupled mVenus reporter gene (Fig. 1C). The plasmid library was transformed to Top10 *E. coli* cells, plated and single colonies, each harbouring a unique spacer, were inoculated in 96-well plates. For each spacer length, 96 clones were analysed in a plate reader. In accordance with transcriptomics data of endogenous promoters, highest average promoter strength was achieved with 17 bp (Fig. 1D). Closely followed by the 18bp spacer. High levels of gene expression were observed in the range of 15bp to 19bp. For longer spacer sequences, a significantly lower moderate level of gene expression was detected (Fig. S1), indicating a suboptimal access of RNAP to the −35 region. For shorter than 15 bp spacers, the expression levels further dropped which may be caused by the limited flexibility of $\sigma {}$^70^ to access the $-10$ and −35 region in parallel. However, promoter activity was still above background levels determined by a random sequence library replacing the promoter region including $-10$ and $-35$ (Fig. 1D ’c’ column). Irrespective of the spacer length, the sequence composition caused an almost 10-fold variation in promoter strength.

### 5’-TG-3’ motifs within the spacer sequence improve RNAP binding and can act as RNAP brakes

**Figure 2:**
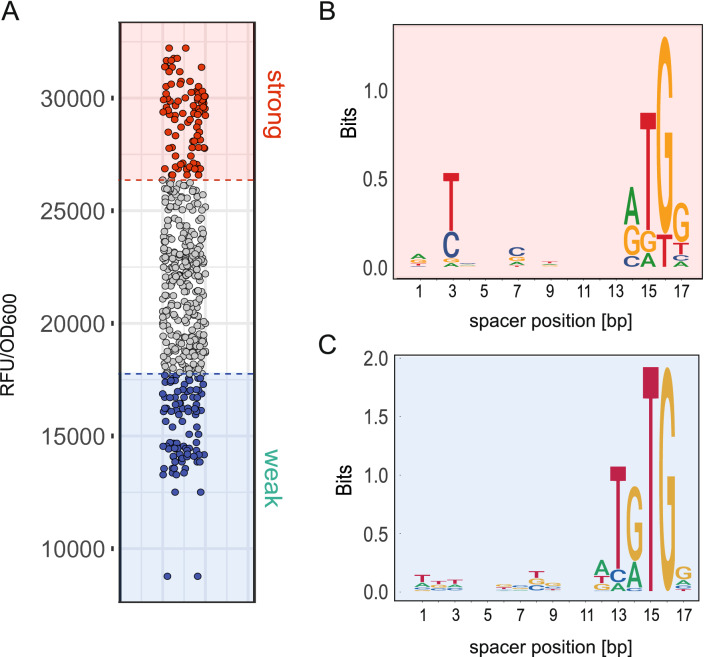
Selection for weak promoters with optimal spacing. **(A)** Selection of the two groups for sequence analysis. Weakest and strongest 17bp spacer sent for sequencing are depicted in blue and red, respectively. **(B)** Sequence logo of the strong promoter group. The relative sizes of the letters indicate their frequency in the sequences. The total height of the letters depicts the information content of the position, in bits reflecting the degree of conservation. **(C)** sequence logo of the weak promoter group.

The 17 bp spacer is the most abundant endogenous spacer of all $\sigma {}$^70^ promoters in *E. coli* (Fig. 1A). In contrast to endogenous promoters, in the synthetic promoter library, the 16 bp spacer was on average as strong as the optimal 17 bp spacer promoters (Fig. 1B,D). In addition, considering individual promoter strengths, an upper bound of expression strength was observed in 16 bp, 17 bp and 18 bp spacer libraries. Hence, what may be causing this discrepancy? A new library of 17 bp promoters was constructed and expression strength of the first library was confirmed. Both libraries were pooled for further analysis. To test how spacer sequence influences promoter strength, the library was divided into three groups: the least active 48 (weak), the most active 48 (strong), and all in between. (Fig. 2A). The weak and strong promoter groups were sequenced and its motif composition was extracted. Analysis of melting energy, bendability and GC-content, revealed no significant difference between the two groups. Neither analysis of the overall mean values nor a position-dependent approach of bendability scores and melting energy revealed a significant distinction. Surprisingly, both groups were enriched in a 5’-TG-3’ motif at spacer location 15-16 (Fig. 2B,C).

**Figure 3:**
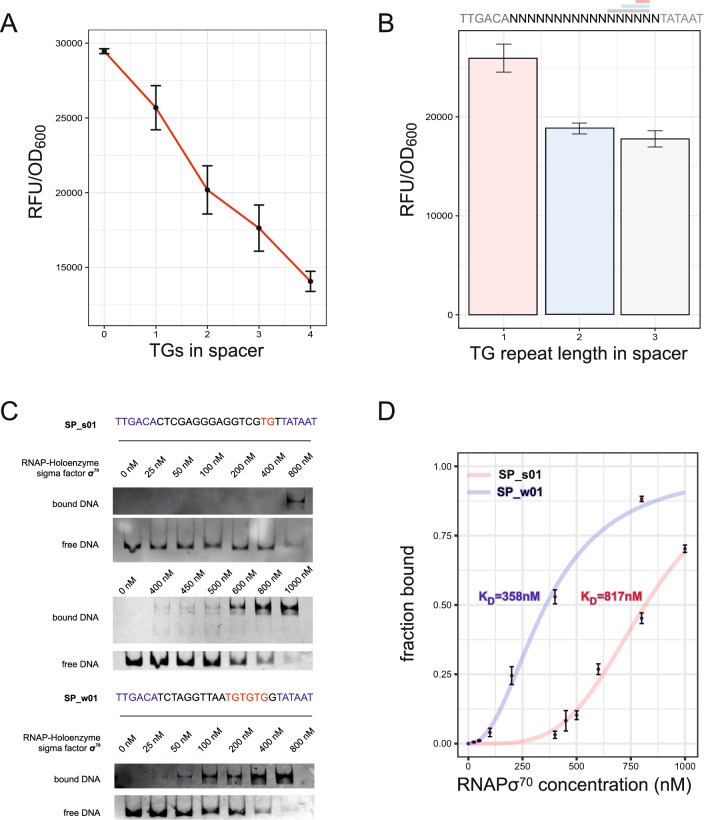
Impact of TG motifs on promoter strength. **(A)** Relation of the number of TGs in the spacer region and the promoter strength. **(B)** Library analysis with different lengths of TG stretches at the extended $-10$ position. Red and blue reference to the strong and weak groups with a similar sequence composition. **(C)** EMSA RNAP$\sigma {}$^70^ binding analysis of the strongest promoter SP_s01 and weakest promoter SP_w01 of the 17 bp spacer screen under selective conditions for weak promoter strength. Vertical space between shifted bands was cropped indicated by a white space. **(D)** EMSA statistics of four replicates for SP_s01 and SP_w01. Error bars indicate standard error of four independent replicates.

This motif is found in so-called extended $-10$ promoter regions. The strong promoters contained this motif in 75 % of sequences, whereas 100 % of promoter sequences in the weak group displayed this motif at spacer position 15-16. Furthermore, the weak group showed significant enrichment not only for one 5’-TG-3’ on this position (Fig. 2B) but for a tandem 5’-TG-3’, leading to a 5’-TGTG-3’-motif. In addition to the obvious tandem enrichment of 5’-TG-3’ at the positions 13-16, spacers with additional 5’-TG-3’ motifs in the spacer sequence were found. Although no position dependent pattern was apparent (Fig. 2B,C), the number of 5’-TG-3’ motifs had a clear influence on promoter activity (Fig. 3A and Fig. S3). In both the weak and strong group combined, the 5’-TG-3’-motif was in more than $90 \%$ of the sequenced promoters on position 15 and 16, an extreme enrichment. A separate library with fixed 5’-TG-3’, 5’-TGTG-3’ and 5’-TGTGTG-3’ motifs at position 16 confirmed that the tandem 5’-TGTG-3’ increased the damping of the promoter compared to the classic $-10$ extended region (Fig. 3B). Longer tandem 5’-TG-3’ motifs added little to the impact. The enrichment of the 5’-TG-3’ motifs at the extended $-10$ region and elsewhere in the spacer sequence raised the question, what might have caused this selection. Cloning of the spacer library without a promoter context showed no bias in library sequence composition. Colony counts of the spacer libraries revealed an 80% lower yield of colonies with 17bp spacers compared to suboptimal 15bp spacers confirming a selective pressure. Test sequencing of 10 15bp spacers showed no enrichment of TG-motifs (0 ocurrances in the extended $-10$ region). Hence, selection due to a toxic effect was apparent. Due to its metabolic costs, overexpression of proteins can be a burden to the cell or interactions of the protein itself with other components of the cell^41^. For GFP, almost identical to mVenus, the limit is about 15 % of all proteins^42^. Hence, the strong 17bp-spaced promoters may have caused a protein burden. Consequently, protein burden was lowered by reduction of the translation rate of mVenus by a weaker RBS. In case of a protein burden, promoters previously selected against, should now be part of the new library. Changing the RBS resulted in a 30-fold reduction of the fluorescence signal (Fig. S5). For a reference, the strongest promoter of the previous library was cloned with the weaker RBS. Surprisingly, no promoter was stronger than the strongest reference promoter from the previous library. Since a change in RBS does not affect mRNA levels, the toxic effect could be due to RNA-toxicity. In 2018, Mittal et al. described a toxic mRNA effect for the fluorescent protein GFP^43^. Similar to our observations, a weaker RBS had no impact on toxicity of the mRNA sequence. Since GFP and mVenus are highly homologous, RNA toxicity is likely to cause the selection in our libraries for strong promoters with optimal spacing and consensus -10 and -35 sequences. Partial deletion studies of mVenus revealed a rather complex interplay of different sequence parts. Furthermore, we inserted silent mutations to remove internal start codons to exclude internal toxic peptide ORFs. These mutations had no effect on toxicity. In conclusion, similar to Mittal et al., we could not finally clarify the cause of the toxicity. Possible other causes are interference of strong promoters with the resistance cassette or plasmid replication. However, the nature of selection has no impact on the promoter-related findings presented in this study. According to the data analysis, 5’-TG-3’ and 5’-TGTG-3’ motifs reduce promoter strength for optimally spaced promoters to circumvent toxic effects. However, a 5’-TG-3’ motif is known to stabilize RNAP binding^44^. Therefore, increased binding affinity is expected. In a native promoter context, a 5’-TG-3’ motif is found at promoters with a weak $-10$ region. We hypothesised that the increased binding affinity caused by 5’-TG-3’- and 5’-TGTG-3’-motifs in the sequences of the 17 bp spacers, lower the transcription of genes controlled by these promoters. The enhanced binding affinity of the 5’-TG-3’-motif together with the optimal $-10$ and $-35$ region sequences result in a strong interaction that may immobilize RNAP at the promoter. The concept is supported by earlier studies indicating that consensus promoters, yielding high RNAP binding affinity, show reduced promoter activity compared to promoters with slight deviations from the consensus^45^. Following this logic, a tandem arrangement of 5’-TG-3’ could amplify the effect and lead to a more effective entrapment of the RNAP at the promoter. This entrapment would, consequently, lead to lower transcription rates and reduced expression. EMSA tests were performed with the strongest and the weakest promoter from our 17 bp spacer promoter library. Notably, the weakest promoter contained a 5’-TGTGTG-3’ triplet at spacer position 11-16, and the strongest contained a single 5’-TG-3’ at positions 15-16. Testing both sequences by EMSA with RNAP revealed a stronger affinity to the enzyme for the weaker promoter (K$_D$ = 358nM±19nM) (Fig. 3C,D and Fig. S6) by a factor of more than 2 compared to the K$_D$ of the most active promoter (K$_D$ = 817nM±4nM) (Fig. 3C,D) supporting the RNAP entrapment hypothesis.

### Spacer length has a minor effect on DNA supercoiling sensitivity

**Figure 4:**
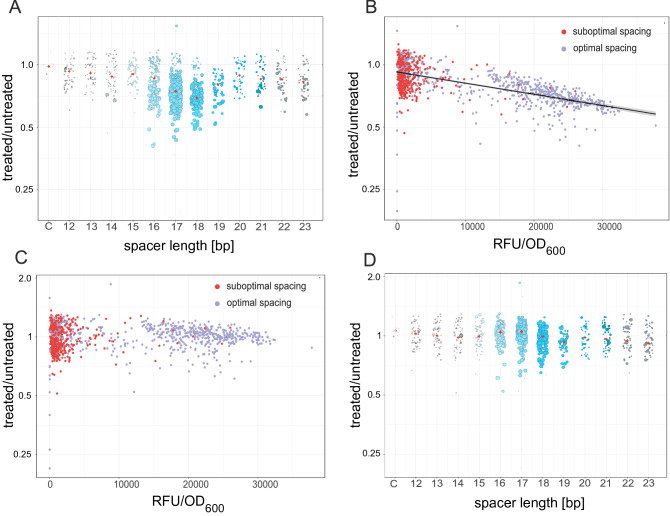
Impact of spacer length and sequence of DNA supercoiling sensitivity. **(A)** Fluorescence ratio of Novobiocin treated samples to untreated samples. Each dot represents a separate clone. The size of the dot indicates its promoter strength in the untreated sample. Blue colors mark spacer lengths also observed in native *Escherichia coli* promoters. Red dots indicate the average for each spacer. **(B)** Relationship of promoter strength and supercoiling sensitivity. Optimal spacers (15–19 bp) are indicated by a blue color. Suboptimal spacers (12–15 and 19–23) are indicated by a red color. Best fit regression line is shown in black. **(C)** Promoter strength bias of figure B is removed by subtracting the regression line. Optimal spacers (15–19 bp) are indicated by a purple color. Suboptimal spacers (12–14 and 20–23) are indicated by a red color. **(D)** The corrected data of (**C**) is used to recompute figure (**A**).

Previous studies of single native promoters indicated that there might be an influence of the spacer to DNA supercoiling sensitivity^35,46^. However, due to the nature of these single base deletion and substitution studies, it is unknown whether the observed effect is due only to the spacing, or whether the sequence of these spacers, e.g. motifs or certain bases, may play a role. To test whether the spacer was mediating supercoiling sensitivity in this screen, previously built promoter libraries were studied under DNA-relaxing conditions by Novobiocin treatment. Novobiocin alters the homeostasis of DNA supercoiling in the cell by inhibition of Gyrase and thus shifts negative DNA supercoiling levels towards relaxation. Previous transcriptomics studies used short harsh and consequently lethal treatments to avoid secondary effects. Though avoiding secondary effects, such harsh conditions do not reflect physiological conditions, a promoter is exposed to in living cells and renders time-resolved expression studies with altered DNA supercoiling levels impossible. To circumvent this shortcoming, we developed a mild treatment procedure to investigate long-term promoter activity in living cells. The previous promoter libraries were first mirrored onto two 96-well plates and added a non-inhibitory concentration (17 $\upmu$g/mL) of novobiocin to one of the duplicates. Then, cells were grown overnight for 15 h. At the applied concentrations, no growth defect could be detected throughout the growth cycle (Fig. 6C,D). Analysis of the topoisomer distribution of pUC18 plasmids using high-resolution gel electrophoresis showed a change in superhelical density $\sigma$ of 0.00195 in exponential phase and 0.00156 in stationary phase upon mild treatment (17 $\upmu$g/mL) with Novobiocin (Fig. S2). Compared with the $\Delta \sigma$ of 0.025 between exponential and stationary phase, the treatments impact is about ten times lower than the growth phase dependent variation in superhelical density. Data analysis revealed that in all spacer lengths promoter activity ranged from approximately 50% increase to a 2-fold decrease (Fig. 4A). The 18 bp spacers displayed the sharpest decline followed by 17 bp spacers and 19 bp. There was a tendency for promoters with the suboptimal spacer lengths 12–14 bp and 20–23 bp sequences to show less reduction in expression when treated with Novobiocin (Fig. 4A). This would indicate a relationship between spacer length and DNA supercoiling sensitivity. However, analysis of the relationship of promoter strength and supercoiling sensitivity revealed a strong negative correlation (Spearman correlation coefficient − 0.644) (Fig. 4B). This negative correlation was also observed for each spacer group separately. The general susceptibility of strong promoters for DNA supercoiling originates from Gyrase inhibition leading to a limited potential of positive supercoil removal downstream of the transcription complex and in turn a higher frequency of disrupted RNA elongation processes. However, this process takes effect after promoter escape and is therefore not affecting promoter related parameters. When corrected for the effects of expression strength (Fig. 4C), a minor but non-significant effect of spacer length remained between the different spacer lengths (Fig. 4D). However, a wide spectrum of supercoiling sensitivities remained within each spacer group (Fig. 4C,D). Since the spacer length is constant in each spacer group, the change has to be associated with the spacer sequence composition. The observation of different supercoiling sensitivities within a spacer group could be reproduced for individual clones in several replicates (Fig. 6A,F) and is therefore not due to variations in the measurements.

### GC-content of the spacer sequence modulates DNA supercoiling sensitivity

**Figure 5:**
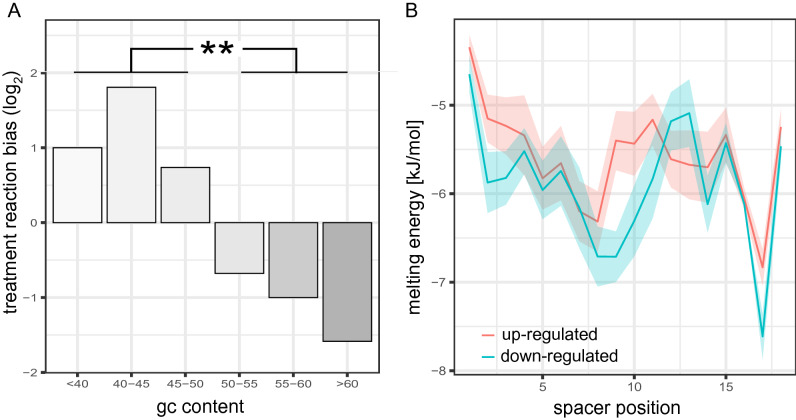
Spacer sequence properties leading to changes in supercoiling sensitivity. **(A)** Relationship of DNA supercoiling sensitivity and spacer GC-content. The number of up-regulated and down-regulated promoters are counted for each GC-content bin after correcting for the expression strength bias. Depicted is the count ratios different GC-content bins. E.g. 2 indicates 2-fold more of up-regulated than down-regulated promoters in the respective bin. **(B)** Melting energy for all dinucleotide steps along the spacer sequence. Mean melting energy and error bars are indicated for promoters with a positive (red) or negative (blue) reaction upon treatment.

**Figure 6:**
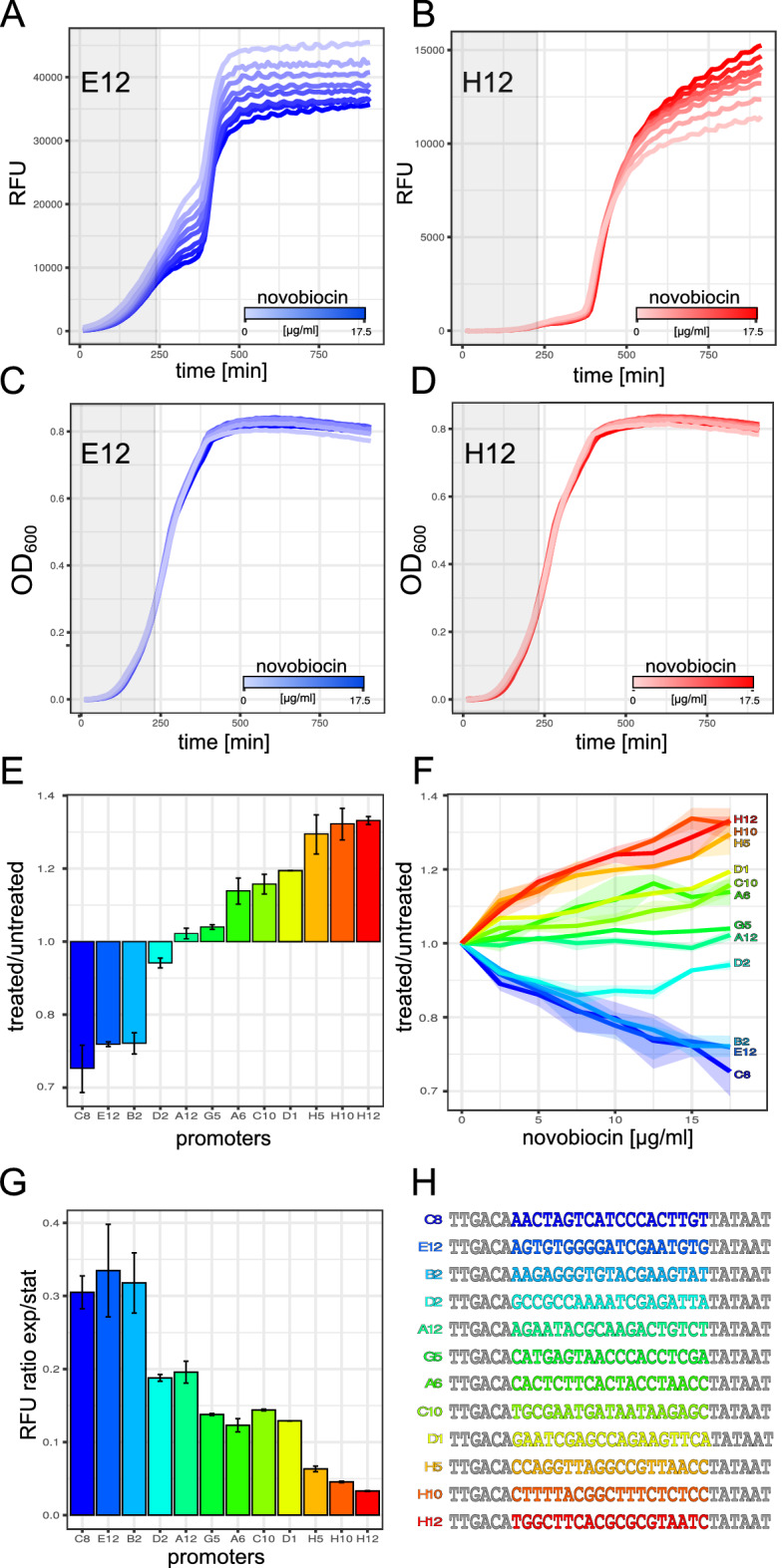
Further analysis of supercoiling sensitivity. **(A)** 5-replicate average mVenus fluorescence signal of promoter E12 with a preference for negative DNA supercoiling. Blue colors with increasing intensity indicate treatment with 0, 2.5, 5, 7.5, 10, 12.5, 15 and 17.5 µg/ml Novobiocin. The grey area spans exponential phase to OD 0.3. Colors facilitate recognition of the promoters in (**A**–**H**). For error bars see (**E**) and (**F**). **(B)** 5-replicate average mVenus fluorescence signal of promoter H12 with a preference for DNA relaxation. Red colors with increasing intensity indicate treatment with 0, 2.5, 5, 7.5, 10, 12.5, 15 and 17.5 µg/ml novobiocin. The grey area spans exponential phase to OD 0.3. Colors facilitate recognition of the promoters in (**A**–**H**). **(C,D)** Corresponding 5-replicate average growth curves of the promoters E12 and H12 (see also **A**,**B**).For error bars see E and F. **(E)** stationary phase fluorescence signal fold change of 12 selected clones under 17.5 µg/ml novobiocin treatment relative to untreated. **(F)** stationary phase fluorescence signal fold change of 12 selected clones treated with various novobiocin concentrations relative to untreated. **(G)** Temporal expression preference of 12 supercoiling sensitive clones without novobiocin treatment (see also light-colored curves in **A**–**D**). Ratio of the amount of expression performed in exponential phase (OD 0.3) compared to stationary phase (last time point). Supercoiling sensitivity strength (see also plot **E**) is indicated by a gradient from blue for a strong preference of negative supercoiling to red for a strong preference of DNA relaxation. **(H)** Sequences of the 12 investigated promoters. The $-35$ and $-10$ regions are indicated in grey. The spacer region is colored according to the color-code in (**A**–**G**).

After the first indication of an impact of spacer sequence composition on DNA supercoiling sensitivity, we split the sequenced groups in positively and negatively reacting clones (above and below 1 in Fig. 4C). After the sequence analysis previously applied to weak and strong promoters, a bias in GC-content was apparent for the two groups (Fig. 5A). Further resolution in GC-content bins shows a gradual decrease of relaxation tolerance with increased GC-content and even positive resonance for AT-rich spacers. The difference in melting energy with varying GC-bias is evenly distributed along the spacer sequence (Fig. 5B). Hence, depending on the promoter GC-content, DNA supercoiling preference could be modulated. The dependence on GC-content and the independence of position within the spacer is consistent with the twist and melt model that requires spacer deformation upon RNAP binding and promoter melting^47–49^. Such deformations are facilitated by reduced melting energies of spacer DNA in the absence of negative twist. Hence, GC-rich spacer sequences require negative DNA supercoiling to propel spacer deformation. Therefore, these spacers react negatively upon DNA relaxation. However, for AT-rich sequences an excess of negative DNA supercoiling favour alternate DNA forms that may be unfavourable for open complex formation or promoter escape^50^. Hence, promoters with AT-rich spacers or spacers with similar properties can be relaxation -tolerant or even increase activity upon relaxation. With only a fraction of the naturally observed DNA supercoiling difference between exponential and stationary phase, synthetic promoters showed significant reactions to mild DNA relaxation. Hence, we investigated the reaction of these promoters to natural growth-phase-dependent DNA supercoiling changes. A set of 12 clones with a variety of DNA supercoiling preferences were selected for time-resolved expression analysis (Fig. 6H). In a first experiment, we further characterized the reaction of the promoters to various levels of DNA supercoiling by gradually altering the concentration of Novobiocin up to the level used for the initial screen (Fig. 6E,F). Two time-resolved representative examples of relaxation intolerance (E12) and preference (H12) are shown in Fig. 6A,B, respectively. Interestingly, not all promoters show a linear relation between Novobiocin concentration and promoter activity. Promoter D2 shows a drop in expression for low concentrations but reverts this tendency for higher concentrations. This could indicate that many promoters have an intrinsic DNA supercoiling optimum not covered by our treatment range nor by harsh treatment. Having a closer look at the expression pattern shows the continuation of expression during stationary phase for H12 (relaxation preference) even without the addition of Novobiocin (opaque red curve in Fig. 6B) and the faster increase of expression during exponential phase for E12 (relaxation intolerant opaque blue curve in Fig. 6A). Analysis of all 12 selected clones revealed a shift of expression towards exponential phase with increasing relaxation intolerance (Fig. 6E–G). The shift towards exponential phase is consistent with the growth phase dependent DNA supercoiling levels in the cell^30^. This observation indicates that the promoter spacer not only has a strong impact on basal promoter strength but also on promoter timing during growth.

## Discussion

Previous studies on promoter architecture suggested an impact of DNA spacer length and sequence on promoter strength and intrinsic supercoiling sensitivity^35^. However, these studies relied on data of native promoters including regulatory sites adapted to a complex genomic context. Furthermore, previous studies made no systematic changes to sequences or spacer length nor screened the full spectrum of sequence and spacer length space, which could provoke misinterpretation of the data. Systematic studies are, therefore, necessary to reveal intrinsic properties of core promoter architecture with high accuracy. In this study, we applied core promoter library analysis and transcriptomics to get an insight into the relationship of core promoter architecture and gene expression. Combining synthetic library design and high-throughput transcriptomics, we could show that the spacer length variety of *E. coli* native promoters covers the full dynamic range of gene expression. This implies a role in long-term adaptation processes. Furthermore, we could show that the sequence composition and length of the spacer has a major impact on promoter strength. In this context, we further characterized the -10 region 5’-TG-3’ extension and found a 5’-TG-3’ tandem repeat motif that reduced promoter activity of consensus promoters. Binding affinity assays revealed increased RNAP binding for tandem 5’-TG-3’ motifs. Interestingly, the enhanced binding of RNAP significantly reduced promoter activity. Earlier studies have shown, that consensus promoters tend to be slightly weaker than promoters with a few deviations from the consensus^45^. Further enhancement of the binding affinity by the reported promoter extension motifs is likely to increase the activity damping effect. Additional 5’-TG-3’ sequences, independent of sequence position, further weakened the promoter. The 5’-TG-3’ and 5’-TGTG-3’ at position 15-16 and 13-16 were present in these cases. Therefore, the promoter weakening cannot be due to a weaker binding of RNAP. Therefore, we conclude that the additional 5’-TG-3’s further stabilize RNAP binding. The random positioning suggests a non-specific potentially structural interferrence with the sigma factor. In the context of strong consensus promoters, we observed that enhanced binding affinity caused a decrease in promoter activity by RNAP trapping at the promoter. Especially in evolution studies and in synthetic promoter design, the spacer region of the promoter should be well observed for effects on basal promoter strength. The impact of various spacer parameters on promoter strength may also explain the relatively poor conservation of promoters of orthologous genes in different bacteria. With a large set of possible promoter sequences exhibiting the same expression profile, genetic drift of a promoter sequence is high due to a relatively low selective pressure. A further characterisation of the promoter susceptibility to physical parameters brought new insight in the emergence of DNA supercoiling sensitivitiy, a major regulatory concept throughout the bacterial kingdom and beyond^16,20,21^. The approach of mild Novobiocin treatment allowed for a physiologically plausible study of time-resolved expression under various levels of DNA relaxation and revealed a role of the spacer sequence in temporal expression control via DNA supercoiling. Mild treatment, in this context means a ten times lower change in DNA supercoiling than observed between exponential and stationary phase. With about 50% of the *E. coli* genes being sensitive to DNA supercoiling, this treatment is mild enough to only minimally influence these sensitive chromosomal promoters to keep the system running without major perturbations. Therefore, the reduction of Gyrase activity caused by Novobiocin does not lead to growth defects. Interestingly, even under such mild conditions, significant effects of the spacer sequence on DNA supercoiling sensitivity are present. Extrapolation of fold-changes to Novobiocin concentrations of harsh treatments yield comparable fold-changes (Fig. S4). This supports previous observations under harsh treatment conditions, that DNA supercoiling has major effects under physiological conditions. The mild treatment also revealed individual DNA supercoiling optima for the investigated promoters. This could indicate that the set of DNA supercoiling sensitive promoters is larger than detected by previous harsh methods and ranges in the physiological spectrum of DNA supercoiling. In contrast to previous assumptions, the approach revealed that the promoter spacer length has minor impact on DNA supercoiling sensitivity. This was not expected in the light of earlier studies^35^. However, in this study we used highly standardized core promoters that may differ in its response spectrum from more complex native promoters. This study showed that the GC-content of the spacer seems to play the crucial role in spacer-mediated DNA supercoiling sensitivity. There was no indication of a $-10$ region proximal bias of GC-content in the analysed spacer sequences. Hence, it is likely that the overall structure of the spacer is affected^51^. Since bending and curvature parameters were not significantly involved, DNA deformation or DNA form conversion connected to DNA melting may be involved. In addition, there is evidence from native supercoiling sensitive promoters, that the GC content all over the promoter sequence affects supercoiling sensitivity^25,46^. Therefore, it could also be part of a promoter-wide mechanism. Moreover, a change in GC-content also alters the base composition. These changes may have impact on the phasing of the flanking $-35$ and $-10$ regions^52^. Interestingly, the supercoiling sensitive *gyrA* promoter, analysed in Menzel et al.^46^, combines the presence of a 5’-TGTG-3’ motif and DNA supercoiling sensitivity. Future studies may set a focus on the interplay of promoter features to control DNA supercoiling sensitivity.

## Conclusion

All in all, the study revealed new adjusting screws of evolution to fine-tune promoter activity, promoter timing and DNA supercoiling sensitivity. As shaping factors of chromosome architecture on an evolutionary time scale, this study might pave the road to improve our understanding of chromosome design principles and optimal gene arrangements in synthetic biology and trigger new concepts in systems biology. Furthermore, the promoter timing aspect via DNA supercoiling renders the promoter spacer an interesting element for promoter design, either to exploit or avoid effects of DNA supercoiling in synthetic circuits and pathways.

## Supplementary Information

Supplementary Legends.

Supplementary Figure 1.

Supplementary Figure 2.

Supplementary Figure 3.

Supplementary Figure 4.

Supplementary Figure 5.

Supplementary Figure 6.

Supplementary Figure 7.

Supplementary Figure 8.
