# Supplementary material for: The bacterial promoter spacer modulates promoter strength and timing by length, TG-motifs and DNA supercoiling sensitivity
Source: Sci Rep. 2021 Dec 22;11:24399. doi: 10.1038/s41598-021-03817-4 (PMC8695583; doi:10.1038/s41598-021-03817-4)
Supplement: Supplementary file 1 — Supplementary Legends. [file 41598_2021_3817_MOESM1_ESM.pdf]

Figure S1. Statistical significance of difference of average gene expression between different spacer lengths in the spacer library. Red numbers indicate the spacer length. All empty squares are non-significant differences (t-Test p-value > 0.05). All squares with any type of blue circle indicate a significant difference (t-Test p-value ≤ 0.05, T-test). light blue circles are closer to the significance limit but still significant (see color-code legend).

Figure S2. Novobiocin treatment and its impact on topology. A high resolution chloroquine agarose gel of pUC18 plasmid taken during exponential phase (exp) and stationary phase (stat) of treated (17 µg/ml Novobiocin) and untreated cells (other lanes cropped). The small shift of the topoisomer distribution within the same growth phase by mild novobiocin treatment can be best observed by the increased intensity of the upper bands in exponential phase during treatment and the reduced intensity of the lower bands during stationary phase.

Figure S3. Effect of dinucleotide abundance in the promoter spacer on promoter strength. Missing values, are due to the absence of such promoters in the study.

Figure S4. Extrapolation of the fold change of 12 investigated promoters to novobiocin concentrations used for transcriptomics analysis. The fold change curves up to 17.5 µg/ml novobiocin are experimental data depicted in figure 4 F. Curves from 18 µg/ml novobiocin onward are linear interpolations for promoters with R2 > 0.9 for the interpolation curve and experimental data.

Figure S5. Range of fluorescence of the 17bp spacer library with two different RBS. The RBS used in this study is referred to as ’standard RBS’. For the standard RBS two replicates are shown to illustrate the consistence of the signal range. The weakest and strongest promoter of the first library is indicated by a blue and green color, respectively. Dashed lines indicate the strength of the two promoters.

Figure S6. Uncropped EMSA gels used to determine KD values of weak and strong promoters. Interpolation in figure 3D is based on these gels after imageJ band intensity quantification.

Figure S7. Spacer length distribution of all promoters listed in regulonDB.

Figure S8. 50bp promoter flanking sequence. The -10 and -35 regions are in bold. The spacer sequence is underlined.
