# Supplementary figures and images for: The bacterial promoter spacer modulates promoter strength and timing by length, TG-motifs and DNA supercoiling sensitivity
Source: Sci Rep. 2021 Dec 22;11:24399. doi: 10.1038/s41598-021-03817-4 (PMC8695583; doi:10.1038/s41598-021-03817-4)

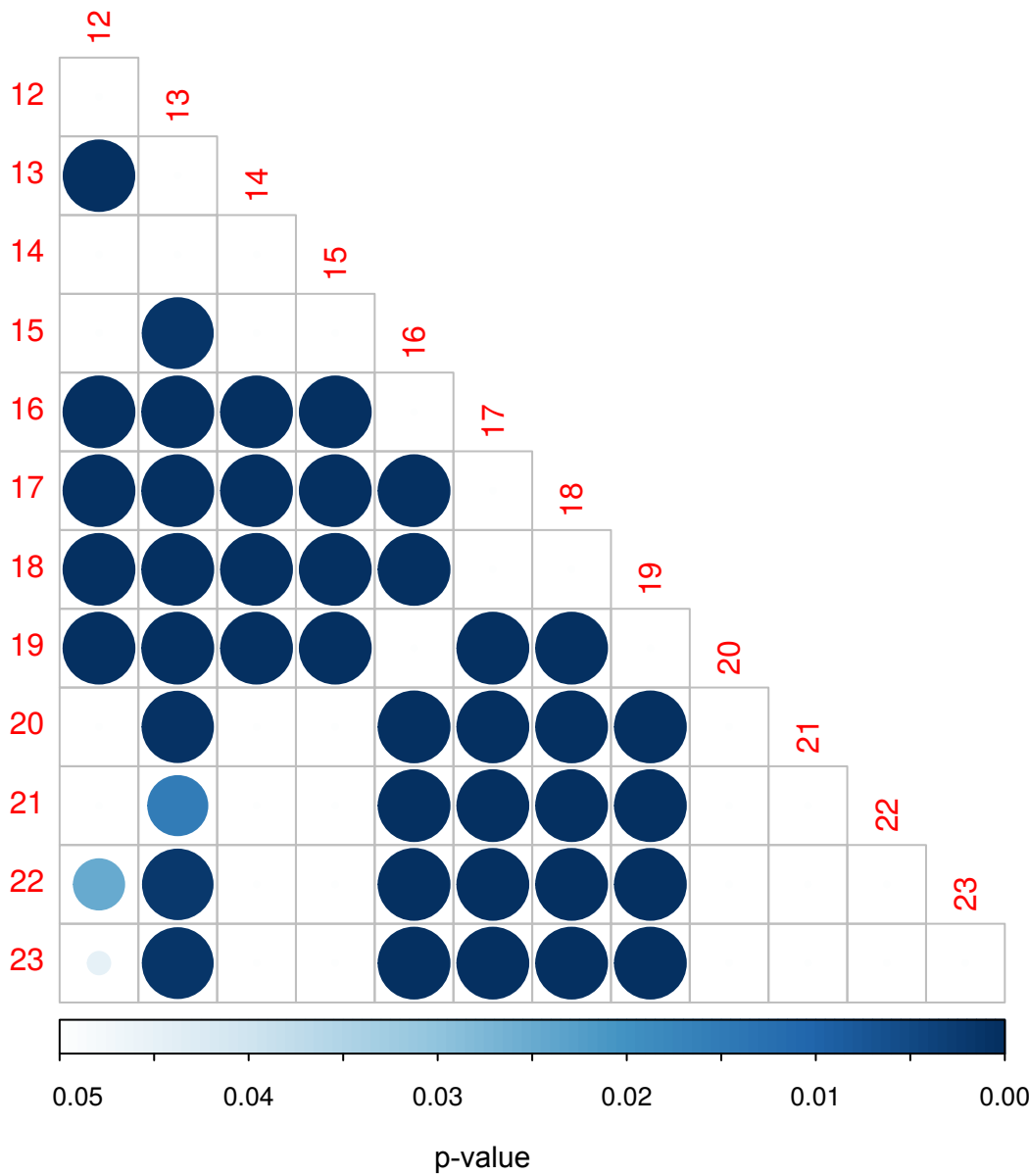

Supplement: Supplementary file 2 — Supplementary Figure 1. [file 41598_2021_3817_MOESM2_ESM.pdf]

exp

stat

17

0

17

0

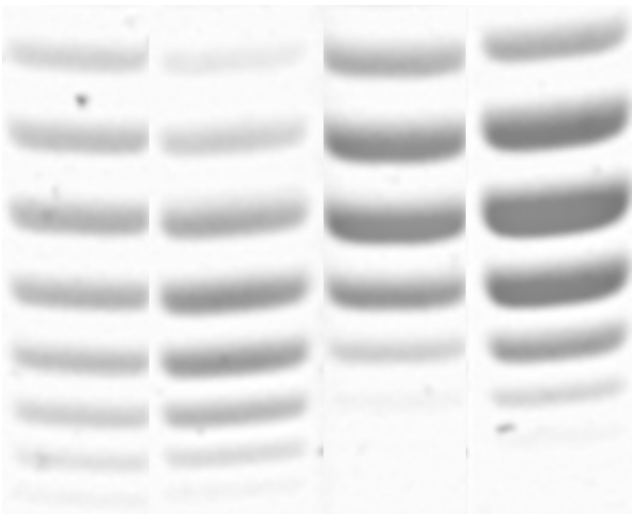

Supplement: Supplementary file 3 — Supplementary Figure 2. [file 41598_2021_3817_MOESM3_ESM.pdf]

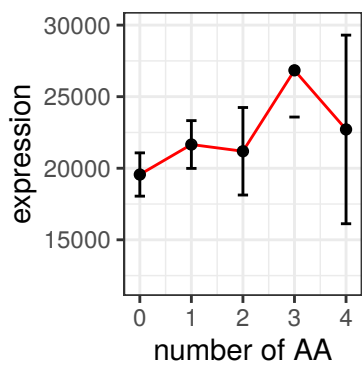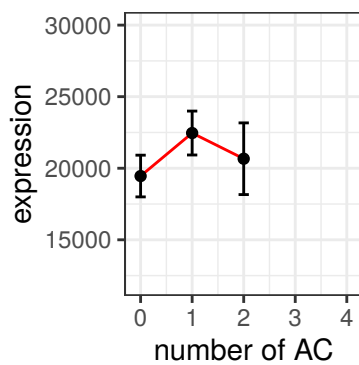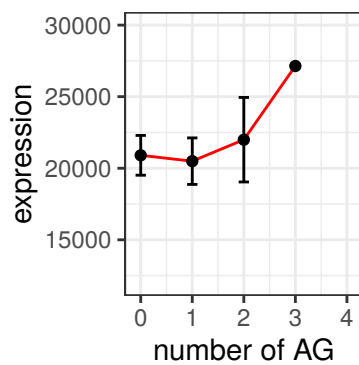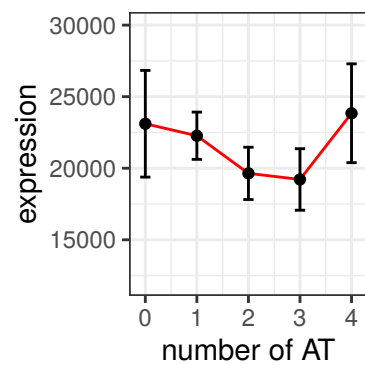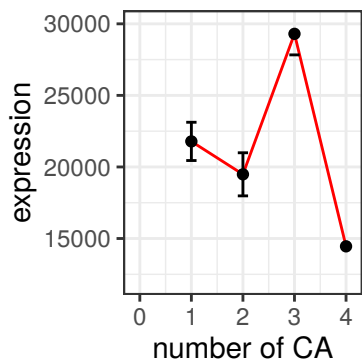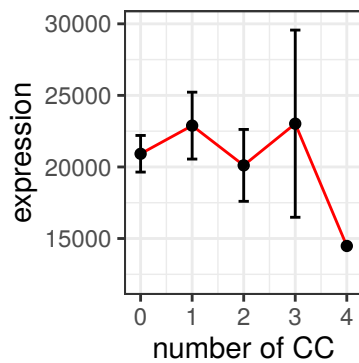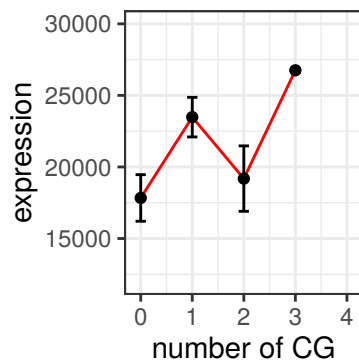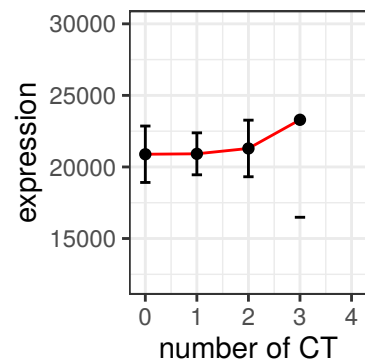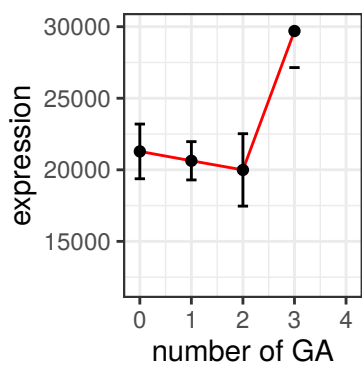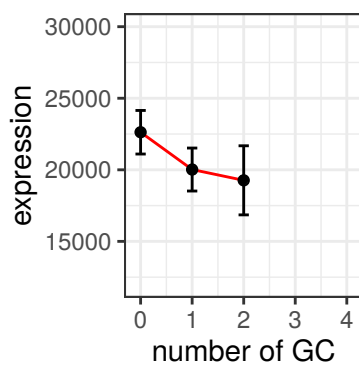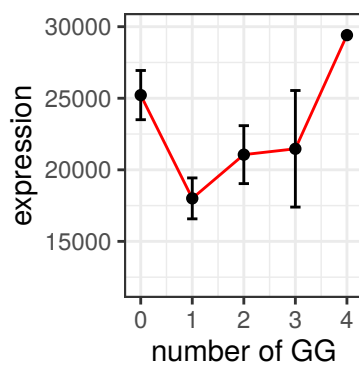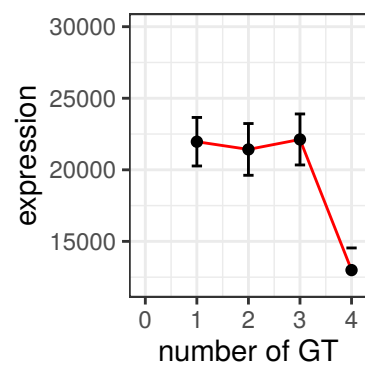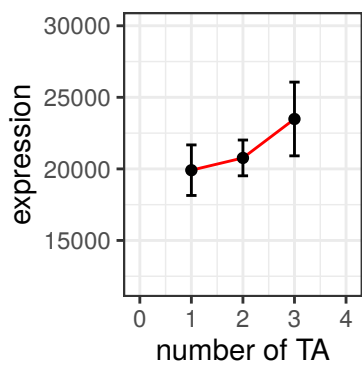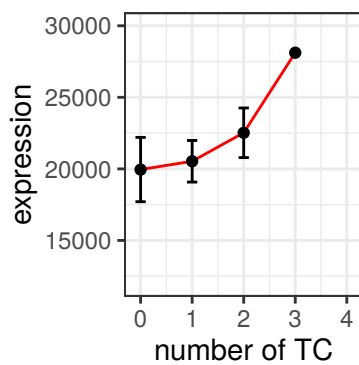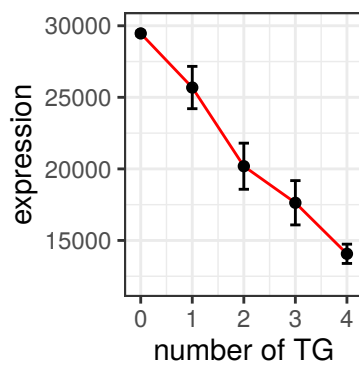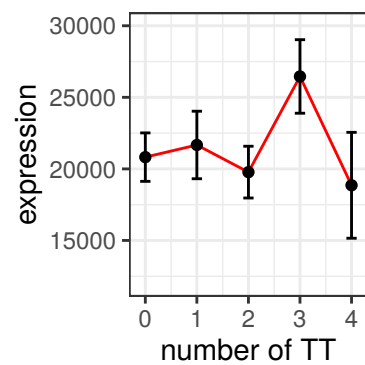

Supplement: Supplementary file 4 — Supplementary Figure 3. [file 41598_2021_3817_MOESM4_ESM.pdf]

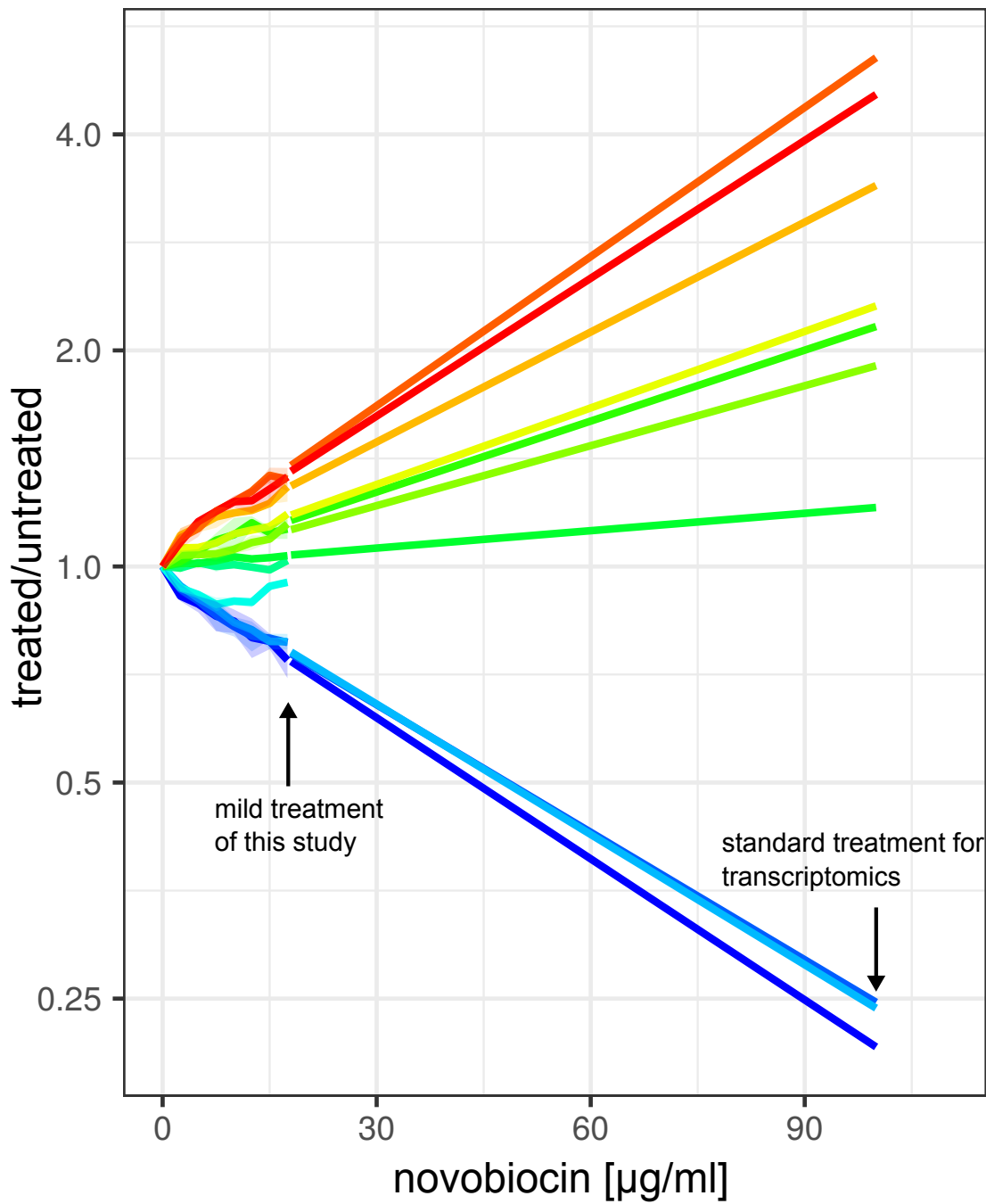

Supplement: Supplementary file 5 — Supplementary Figure 4. [file 41598_2021_3817_MOESM5_ESM.pdf]

number of promoters

400  
300  
200  
100  
0

5 6 7 8 9 10 11 12 13 14 15 16 17 18 19 20 21

spacer length [bp]

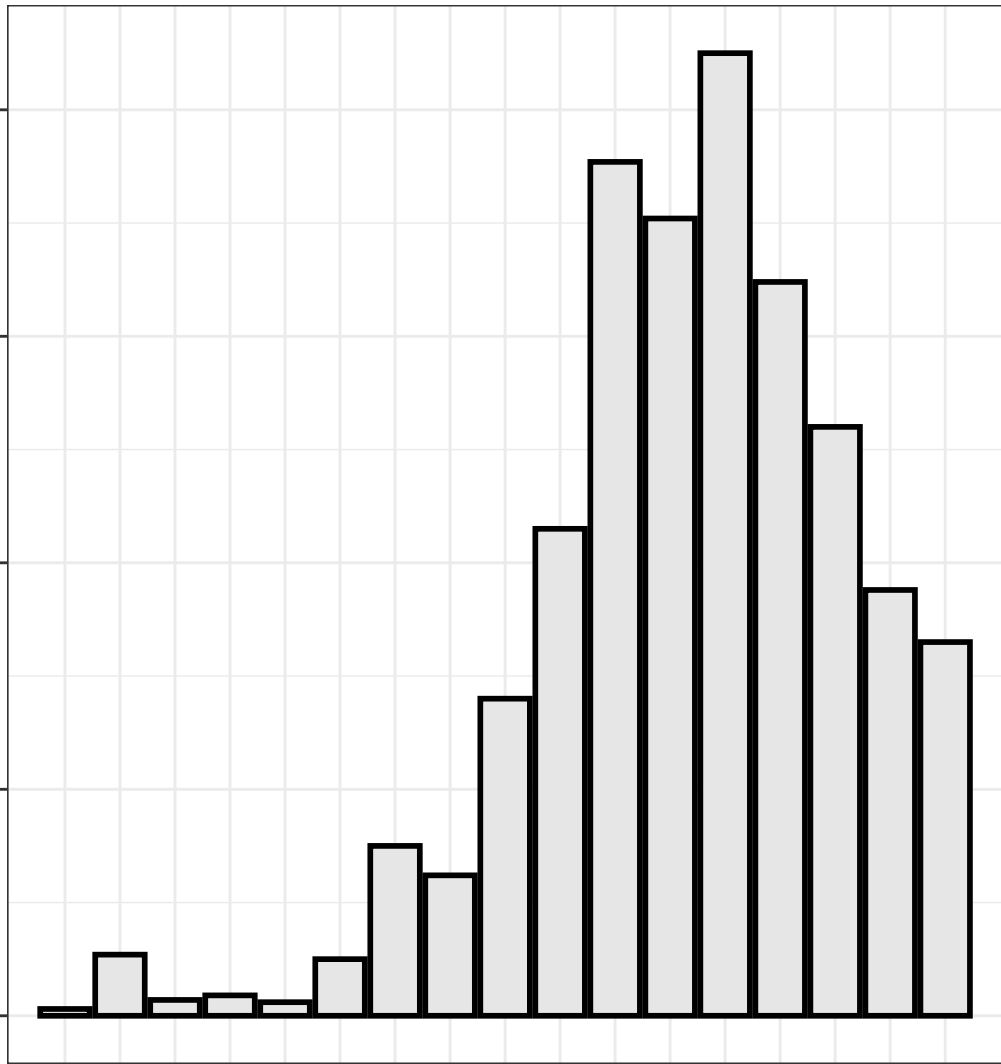

Supplement: Supplementary file 8 — Supplementary Figure 7. [file 41598_2021_3817_MOESM8_ESM.pdf]
