# Supplementary material for: The bacterial promoter spacer modulates promoter strength and timing by length, TG-motifs and DNA supercoiling sensitivity
Source: Sci Rep. 2021 Dec 22;11:24399. doi: 10.1038/s41598-021-03817-4 (PMC8695583; doi:10.1038/s41598-021-03817-4)
Supplement: Supplementary file 6 — Supplementary Figure 5. [file 41598_2021_3817_MOESM6_ESM.pdf]

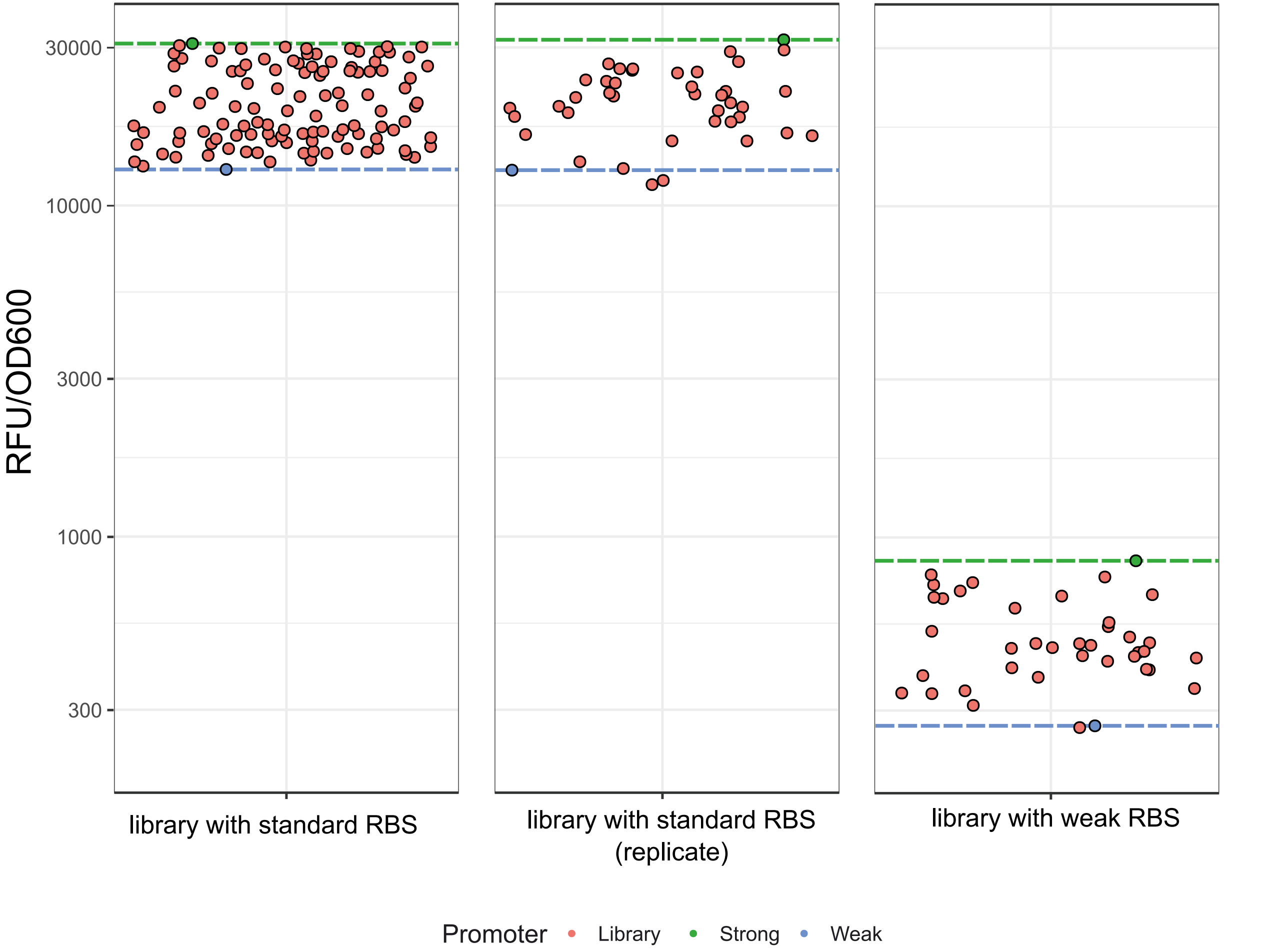

RFU/OD600
30000
10000
3000
1000
300
library with standard RBS
library with standard RBS (replicate)
library with weak RBS
Promoter
Library
Strong
Weak
