# Supplementary material for: The bacterial promoter spacer modulates promoter strength and timing by length, TG-motifs and DNA supercoiling sensitivity
Source: Sci Rep. 2021 Dec 22;11:24399. doi: 10.1038/s41598-021-03817-4 (PMC8695583; doi:10.1038/s41598-021-03817-4)
Supplement: Supplementary file 7 — Supplementary Figure 6. [file 41598_2021_3817_MOESM7_ESM.pdf]

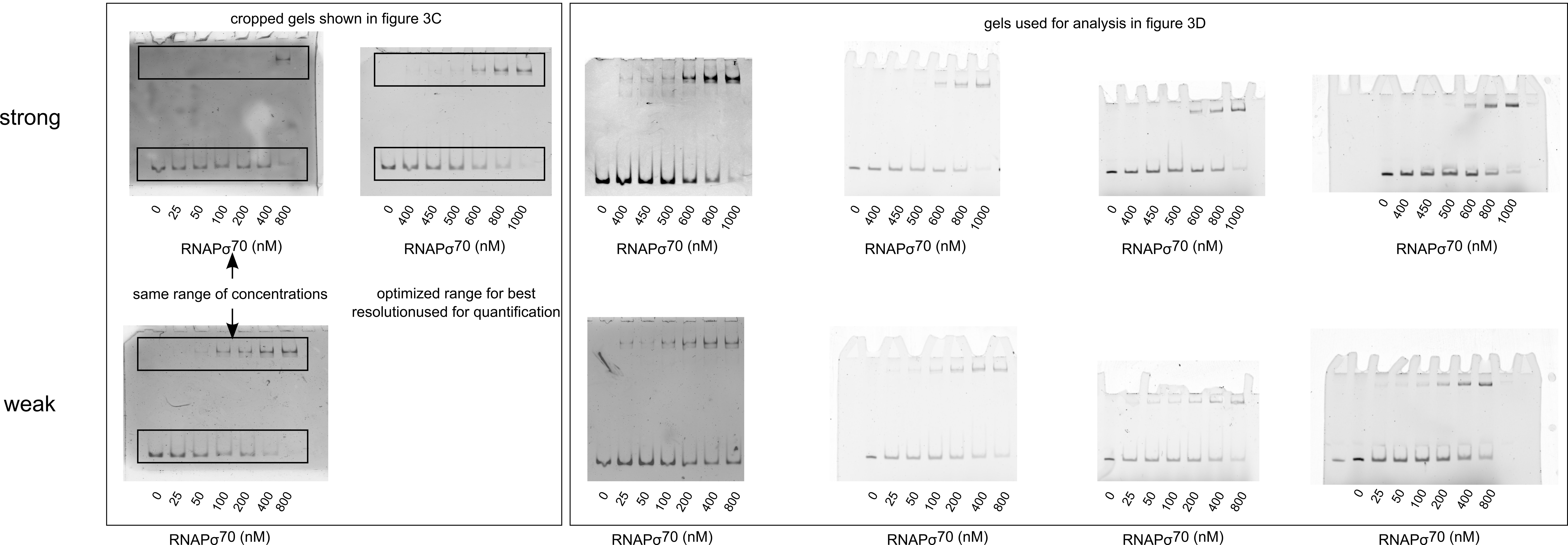

strong
weak
cropped gels shown in figure 3C
gels used for analysis in figure 3D
0 25 50 100 200 400 800
0 400 450 500 600 800 1000
RNAPσ70 (nM)
same range of concentrations
optimized range for best resolutionused for quantification
