# Supplementary material for: The bacterial promoter spacer modulates promoter strength and timing by length, TG-motifs and DNA supercoiling sensitivity
Source: Sci Rep. 2021 Dec 22;11:24399. doi: 10.1038/s41598-021-03817-4 (PMC8695583; doi:10.1038/s41598-021-03817-4)
Supplement: Supplementary file 9 — Supplementary Figure 8. [file 41598_2021_3817_MOESM9_ESM.pdf]

spacer library flanking regions

GGGGTTTTTTGCGGGTAGCGACCGAGTGAGCTAGCGGCGAAG
ACATGGAG**TTGACA**<u>NNNNNNNNNNNNNNNNN</u>**TATAAT**TAGCTA
CGATTACTAGGTCTTCGACGCTCTTTAACAATTTATCAGATCCA
